# Postglacial Fringing-Reef to Barrier-Reef conversion on Tahiti links Darwin's reef types

**DOI:** 10.1038/srep04997

**Published:** 2014-05-21

**Authors:** Paul Blanchon, Marian Granados-Corea, Elizabeth Abbey, Juan C. Braga, Colin Braithwaite, David M. Kennedy, Tom Spencer, Jody M. Webster, Colin D. Woodroffe

**Affiliations:** 1 Unidad de Sistemas Arrecifales (Puerto Morelos), Instituto de Ciencias del Mar y Limnologia, Universidad Nacional Autonoma de México, A.P. 1152, Cancun, Quintana Roo, C.P. 77500, Mexico; 2 Unidad de Sistemas Arrecifales (Puerto Morelos), Posgrado en Ciencias del Mar y Limnologia, Universidad Nacional Autonoma de México, A.P. 1152, Cancun, Quintana Roo, C.P. 77500, Mexico; 3 School of Geosciences, University of Sydney, NSW 2006, Australia; 4 Departamento de Estratigrafia y Paleontologia, Universidad de Granada, Campus Fuentenueva, 18002 Granada, Spain; 5 School of Geographical and Earth Sciences, University of Glasgow, Glasgow G12 8QQ, UK; 6 Department of Resource Management and Geography, The University of Melbourne, Parkville Vic 3010, Australia; 7 Cambridge Coastal Research Unit, Department of Geography, University of Cambridge, Downing Place, Cambridge CB2 3EN, UK; 8 Geocoastal Research Group, School of Geosciences, The University of Sydney, NSW 2006, Australia; 9 School of Earth and Environmental Sciences, University of Wollongong, NSW 2522, Australia

## Abstract

In 1842 Charles Darwin claimed that vertical growth on a subsiding foundation caused fringing reefs to transform into barrier reefs then atolls. Yet historically no transition between reef types has been discovered and they are widely considered to develop independently from antecedent foundations during glacio-eustatic sea-level rise. Here we reconstruct reef development from cores recovered by IODP Expedition 310 to Tahiti, and show that a fringing reef retreated upslope during postglacial sea-level rise and transformed into a barrier reef when it encountered a Pleistocene reef-flat platform. The reef became stranded on the platform edge, creating a lagoon that isolated it from coastal sediment and facilitated a switch to a faster-growing coral assemblage dominated by acroporids. The switch increased the reef's accretion rate, allowing it to keep pace with rising sea level, and transform into a barrier reef. This retreat mechanism not only links Darwin's reef types, but explains the re-occupation of reefs during Pleistocene glacio-eustacy.

Darwin's coral-reef hypothesis, that fringing reefs transform first into barrier reefs and then atolls during subsidence, came into focus in November 1835 when HMS Beagle visited the South Pacific island of Tahiti^1^. Climbing its volcanic slopes, Darwin looked across to neighboring Moorea, and saw a volcanic island encircled by its barrier reef. He surmised that if this reef grew vertically and maintained itself at sea level (SL), then all that was required to convert a barrier reef into an atoll was subsidence of the central island. But the presumption of static SL during subsidence came into question following the discovery of the Pleistocene glaciations^2^, and led Daly^3^ to suggest that reef types were not genetically related, but formed independently from differential wave-erosion of volcanic platforms during glacially-lowered SL. However both ideas predicted single episodes of vertical reef growth in response to relative SL change (Supplementary Fig. S1 online).

Drilling to test these hypotheses subsequently showed that atolls were not formed by uninterrupted accretion of a single reef unit, but by stacked units formed during SL highstands, separated by laterally extensive subaerial-erosion surfaces formed during lowstands^4^. In most cases, Holocene reef units beneath reef flats are only 10-20 m thick, and overlie as many as 5 older highstand reef units of similar thicknesses^5^,^6^. Beneath this thin Pleistocene sequence are hundreds of metres of Cenozoic reef limestones, punctuated by similar exposure surfaces, and eventually a volcanic foundation^7^. Clearly, atolls are not formed during a single episode of vertical reef growth but have complex thermotectonic and eustatic histories that reflect their long plate-tectonic migration^8^.

Although data from drilling confirmed Darwin's postulate that atolls had subsided^9^, they were insufficient to confirm the link between reef types. As Vaughan^10^ stated (p. 325) “Although the theoretic possibility of the conversion of a fringing reef into a barrier and a barrier reef into an atoll may not be denied, no instance of such a conversion has yet been discovered.” This apparent lack of evidence beneath modern atolls led to further claims that there was no link between reef types, and that karstification of the limestone substrate during glacial lowstands was responsible for the raised rims and other morphological features of atolls and barrier reefs^11^,^12^ (Supplementary Fig. S1 online).

Evidence supporting a link between reef types, however, had already started to emerge from last-Interglacial reef-terraces on the Huon Peninsula, where fringing and barrier reefs developed over apparently simple sloping substrates with no apparent karst rims^13^. These fossil reefs were substantial structures, up to 50 m thick, but their developmental history was obscured by imprecise dating, and it was unclear if the transition between reef types occurred during a single episode of reef accretion^14^. Although the Huon data seemed to confirm that the link between reef types was more than just a “theoretic possibility”, it was still unclear why fringing reefs should develop into barriers and not simply retreat upslope tracking SL rise^15^. Their apparent formation on simple slopes implies that some process fixes their position during SL rise and causes them to accrete vertically.

Here we present evidence supporting the conversion of a fringing to a barrier reef on Tahiti, where Darwin first visualized his reef-types hypothesis. By integrating new sedimentological and paleo-depth data with coral and coralline-algal assemblages, we reconstruct the structure and shallow zonation of the postglacial reef sequence recovered in drill-cores from the north (Tiarei) and south (Maara) coasts of Tahiti during the Integrated Ocean Drilling Program (IODP) Expedition 310. Using published dates, we calculate rates of vertical accretion and determine reef development between 15 and 10 ka. When combined with cores from the modern barrier reef, these data provide a complete picture of postglacial reef development and allow us to identify, for the first time, a viable mechanism controlling the conversion between reef types during glacio-eustatic sea level cycles.

Tahiti, a slowly subsiding high volcanic island, is perhaps the ideal site at which to identify conversion between reef types because its modern reef and fore-reef slope have been extensively sampled by drilling, and rigorously dated in order to reconstruct postglacial SL history^16^,^17^. Boreholes drilled along the modern barrier-reef fronting Papeete Harbour on the northwest coast (Fig. 1) show that reef development initiated 14 ka ago at a depth of 90 m^16^, and consisted of a well-preserved sequence of shallow-water corals, dominated in the upper 65 m by robust, reef-crest colonies of *Acropora* spp^18^,^19^. To reconstruct SL history prior to barrier-reef development, boreholes have also been drilled at sites on the fore-reef slope during IODP-Expedition 310^20^ (Fig. 1). However, reef-framework sequences recovered from these sites do not show the same widespread reef-crest *Acropora* assemblage found in the Papeete barrier-reef^21^,^22^ and the determination of early postglacial SL history has relied on the use of corals with a larger depth range, such as branching *Pocillopora* and massive *Porites* spp^17^,^19^. Furthermore, qualitative analyses of coral assemblages show that acroporids were absent or had low abundance prior to 12 ka^21^,^22^.

## Results

### Tahiti's Early Postglacial Reef Sequence

We characterized detrital and framework units in all cores recovered from 13 drill sites at Tiarei (15 holes) and 7 drill sites at Maara (20 holes) during IODP-Expedition 310 (Methods). At the base of each site we identified a 1–3 m thick fringing-reef unit characterized by a densely packed framework of heavily bioeroded, shallow-water corals with a distinctive interstitial matrix of mixed skeletal/volcanic sand-and-gravel. The coral assemblage is dominated by small encrusting colonies of either irregular *Montipora* sheets, or ridged or compact-branched forms of *Pocillopora* sp*.* with close interbranch spacing and large basal attachments (Fig. 2). In the case of *Pocillopora*, this combination of morphological traits is only found in colonies that develop in near-sea-surface conditions affected by wave turbulence^23^,^24^ and is reported to reflect mechanical adaptation to surf-zone conditions^25^,^26^. Based on average wave heights of 2.0–2.5 m reported^27^ from the south coast of Tahiti, we estimate that these surf-adapted corals grew within ~1–2 m of mean low water.

The fringing-reef unit and its surf-adapted corals is in direct contact with the sloping Pleistocene foundation, and the elevation overlap between sites shows that it forms two thin layers: a downslope layer between sites 16 and 12A, ranging in depth from 123–107 m, and an upslope layer between sites 5 and 7, ranging in depth from 94–85 m (Fig. 3). However, the fringing-reef layer does not extend to the Papeete barrier-reef sites, nor to Maraa site 17A. The ^230^Th age of corals in these layers decreases progressively upslope, signifying that substrate colonisation was time-transgressive. The lower layer formed over a ~1600 year interval between 16.13 ka (site 9) and 14.51 ka (site 15). This age decrease is reflected in the upper layer, but it is only dated at site 23, where it formed after 14.31 ka (Fig. 3). Time-transgressive substrate colonisation by surf-adapted corals implies that these layers were produced during the upslope retreat of a shore-attached fringing reef during rising SL. The break in the downslope and upslope layers between sites 12A and 5, corresponds to the drowning and re-establishment of this fringing reef during Meltwater Pulse 1a (Mwp-1a), the first acceleration in Postglacial SL rise^17^.

To determine if a seaward barrier reef was present when the fringing reef was retreating upslope, we reconstruct the paleo-water depth of the overlying framework units at each site. This reconstruction compares the elevation of corals in these units, with the highest coeval corals in all cores, including the Papeete barrier-reef (Methods). It shows that, rather than reflecting a gradual deepening-up sequence, framework units overlying the fringing reef deepen abruptly at all sites (Fig. 3). At Tiarei site 23 for example, a thin layer of encrusting agariciids directly overlying the fringing-reef unit at 91 m, has the same age as robust tabular acroporids at 85 m in Papeete site 7–8 (Fig. 3). The elevation difference implies that the agariciids had a minimum paleowater depth of 6 m, and requires that there was a zone of non-deposition in front of the Tiarei fringing reef between 2–6 m (assuming the surf-adapted corals of the fringing reef are restricted to 0–2 m of water). At Maraa site 15, the paleo-depth of corals directly overlying the fringing-reef unit is greater. Massive *Porites* at 107 m, for example, has the same age as robust acroporids at 87.5 m in Papeete site 7–8, showing that it had a minimum paleo-depth of ~20 m (Fig. 3). Similarly, this requires that there was a zone of non-deposition between 2–20 m in the Maraa reef front.

Reconstruction of paleo-water depths at all Maraa sites with radiometric ages shows that the non-depositional zone in the reef front consistently extends to depths of 15–20 m, and that this zone is represented in core by a small gap in the sequence filled with a poorly-recovered sediment interval (Fig. 3). At Tiarei sites, this non-depositional zone is smaller but varies between depths of 5–20 m, implying that a shallow reef-front framework was present, but only developed in patches (Supplementary Fig. S2 online). The absence of framework in the shallow reef-front at Maraa sites, and the patchy distribution at Tiarei, combined with the deepening-up succession in all cores, confirms that no barrier reef was present in front of the fringing reef between ~16 ka and ~14 ka. Thus, the early postglacial reef development around Tahiti consisted of a shore-attached fringing-reef system that was exposed to high-energy open-ocean conditions.

In order to identify the potential of the fringing-reef unit to keep pace with rising SL, we compare its rate of vertical accretion with framework units at other sites. For each unit, vertical accretion is calculated between adjacent age values, providing a measure of both transient and average accretion (Methods). Transient accretion rates for the fringing-reef unit range from 1.2 mm yr^−1^ (site 9) to 5.0 mm yr^−1^ (site 23) and average 3.6 mm yr^−1^ (Fig. 4). By contrast, the reef-crest *Acropora* unit between 65–50 m in Papeete site 7–8, has an average accretion rate of ~16 mm yr^−1^, but shows transient rates of up to 49 mm yr^−1^. At Tiarei site 23, an open-branch *Porites* unit shows an average accretion of 10.1 mm yr^−1^ with transient rates of 23.3 mm yr^−1^ (Fig. 4). These data illustrate that the fringing-reef unit has lower rates of accretion than either the dominant reef-building units overlying it at the same sites, or the barrier-reef unit at Papeete. Furthermore, this low accretion rate is independent of the rate at which SL was rising, with the fringing-reef unit having similar rates during the slow, early postglacial SL rise prior to Mwp-1a (Tiarei site 9 and Maraa site 15), as during the rapid SL rise immediately following (Tiarei site 23).

Possible causes of low vertical-accretion rates in the fringing-reef unit become clear on examination of its biotic and sedimentary characteristics (Fig. 2). The unit's coral assemblage consists of small (<25 cm), bioeroded colonies, implying high rates of mortality^28^. In addition, it typically lacks fast-growing acroporid corals and thick coralline-algal crusts, both of which are key components for significant reef accretion in shallow reef settings^29^. Acroporids and crustose coralline algae, however, are known to be inhibited by high sediment flux^30^,^31^. Sediment inhibition is also consistent with the presence of skeletal and alluvial sediment filling the interstices between corals (Fig. 2), and forming mostly unrecovered deposits immediately overlying the unit (see partial recovery at Maraa site 15 and Tiarei site 21). Combined, these data imply that the fringing reef experienced a high sediment flux, likely mediated by near-shore wave suspension, that excluded fast-growing acroporids and binding coralline-algal crusts thereby reducing the vertical-accretion potential, and forcing it to retreat upslope in order to maintain its surf-zone position.

### Fringing- to barrier-reef transition

To determine when the fringing reef stopped retreating upslope and transformed into a vertically-accreting barrier reef, we compare the unit's stratigraphic relations and substrate types. The last dated fringing-reef unit occurs at Tiarei site 23, where it was present until 14.27 ka at a depth of 91 m, a little more than 300 years before the Papeete barrier sequence was initiated at 13.93 ka at 90 m^16^. However, at Maraa site 7, an undated fringing-reef unit is present at a shallower depth of 85 m, implying that it was coeval with the formation of the Papeete barrier reef (Fig. 3). Unfortunately, no core data exists for the barrier-reef at Maraa, so the exact timing of the fringing-barrier transition cannot be determined. The transition at Papeete is also difficult to determine because the basal sequence does not consist of robust surf-zone acroporids, which first appear at 12.28 ka at a depth of 65 m in site 9–10 (Fig. 3). As a consequence the fringing- to barrier-reef transition occurred in the 1700 year interval between the last fringing-reef unit at ~14 ka (Maraa site 7), and the first appearance of the Papeete barrier-reef sequence at 12.28 ka.

Importantly, both the Papeete barrier-reef sequence and the fringing-reef unit at Maraa overlie a late Pleistocene reef-flat substrate (Supplementary Fig. S3), which forms the upper part of a conformable sequence that returned ages of ~132–137 ka, suggesting that it formed during the Last Interglacial (MIS-5e) or younger^32^,^33^ (Supplementary Discussion and Fig. S4). This reef-flat substrate forms a horizontal platform at a depth of ~85 m at Papeete site 7–8^19^, Faaa site 19A, (~270 m seaward of the Papeete Barrier), and Maraa site 7 (Fig. 3). Its presence at all three sites implies that it played a key role in halting the upslope retreat of the fringing-reef unit, and facilitated the transition into a fixed barrier-reef sequence. This is because the level or minor reverse slope provided by a reef-flat platform would form a physical barrier to the up-slope retreat of any shore-attached fringing reef during rising SL, as it would force its surf-adapted coral assemblage to retreat into the lower energy and slightly deeper waters of the platform interior. Once the fringing reef reached the edge of the platform therefore, it's upslope retreat would stall, and subsequent SL rise would rapidly create a lagoon behind it.

The development of a lagoon would have had several important consequences: first, the fringing reef would be rapidly isolated from coastal sediment flux as the lagoon width increased by shoreline retreat during SL rise. Second, terrigenous sediment would now be trapped in the lagoon, reducing the sediment flux in the reef-front zone. Both of these effects would reduce the sediment flux that previously inhibited vertical accretion, and allow the fringing-reef and reef-front zone to be colonized by rapid-growing species of *Acropora* and thick crusts of coralline algae. This, in turn, would allow the reef to increase its vertical accretion rate and keep pace with SL rise, transforming it from a fringing reef into a barrier reef.

This lagoonal sediment-trapping conjecture is consistent with taxonomic analysis of reef-front coral and coralline-algal assemblages^21^, which shows an abrupt appearance of acroporids at ~13 ka in Maraa sites (12.94 ka at site 7, 13.16 ka at site 5), with acroporids becoming an abundant component of the assemblage by ~12 ka (Fig. 3). At shallow Tiarei sites, acroporids also make an abrupt appearance at ~13 ka (>12.73 at site 23, and 12.49 ka at site 21), but do not become a dominant component of the reef-front assemblage due to intermittent barrier-reef development on this side of the island and continued high sediment flux (the Tiarei site lies directly seawards of a wide pass in a submerged barrier).

These data imply that the fringing-reef retreat stalled on the Pleistocene reef-flat platform at about the same time as the basal sequence was initiated at Papeete ~14 ka ago. If the appearance of acroporids ~13 ka ago at the Maraa sites signifies that the newly-formed lagoon started to function as a sediment trap, then it apparently took ~1000 years for the sediment flux to reduce sufficiently to allow the fringing reef to start accreting vertically and form a barrier reef at Maraa. However this young barrier may still have been permeable where coastal sediment flux was highest, given that it took a further thousand years for acroporids to dominate reef-front assemblages.

## Discussion

### Linking Darwin's reef types

In Darwin's genetic model of reef types, fringing reefs maintain a fixed position and accrete vertically in response to relative SL rise. However, during glacial-interglacial cycles, this simple mechanism would produce a barrier reef each time SL rose, and lead to the development of multiple reefs at the same location. Data from Tahiti show that, rather than accreting vertically, the postglacial fringing reef was forced to retreat upslope during SL rise due to the exclusion of rapidly-growing acroporids by sedimentation. The transition into a barrier reef seems to have been initiated when the fringing reef encountered a laterally extensive platform which prevented further retreat. Its newly fixed position on the edge of the platform ‘instantaneously’ created a lagoon that trapped sediment, reducing sediment flux, and eventually allowed acroporids to recolonize the reef. This restored its vertical-accretion potential, enabling it to keep-up with rising SL and become a barrier reef.

The key element in the transition between reef types is therefore the presence of a platform with a horizontal or low reverse slope. Evidence from Tahiti implies that this platform was generated by reef-flat development, possibly during the Last Interglacial, although no ages exist for the upper surface of the platform (Supplementary Discussion). Nevertheless, the potential for reef flats to produce wide horizontal platforms during interglacials is well established from Holocene sequences recovered from modern fringing reefs^34^,^35^.

The tendency for fringing reefs to retreat upslope during postglacial SL rise, and to form horizontal platforms during interglacial highstands, provides the key requirements for a genetic link between reef types. In Figure 5, we summarise this link for volcanic islands undergoing slow thermotectonic subsidence. Reef development starts (or is reset) during glacial lowstands. On young volcanic islands, the steep eroding flanks keep fringing reefs close to shore, and high coastal sediment flux suppresses their accretion rate. As a result, during postglacial SL rise, they retreat upslope tracking the transgression and produce a thin diachronous unit. During the ensuing interglacial, the decreasing rate of SL rise is eventually matched and exceeded by the reef's accretion rate, and it can advance seawards producing a wide reef-flat platform (Fig. 5).

This first cycle of fringing-reef development is terminated by SL fall during glacial onset and, over the next ~100 ka, terrestrial erosion and thermotectonic subsidence continue. The rate of subsidence will control the form of subsequent postglacial reef development. Fringing reefs will only develop on islands where the subsidence rate is greater than the amplitude of the SL cycle, because the reef-flat platform that developed during the first cycle will be displaced below the glacial lowstand (e.g., rates >1.2 mm yr^−1^ for the last 120 m lowstand). However, on islands where subsidence cannot displace this platform below the SL lowstand (e.g., rates <1.2 mm yr^−1^), a barrier reef will develop. This is because the second-cycle fringing-reef will retreat upslope with the postglacial rise until it encounters the subsided platform (Fig. 5). The reverse slope of the platform will prevent further retreat and stabilize the fringing reef on its outer edge, initiating barrier-reef development. Thus, as in the case of Tahiti where subsidence is less than the amplitude of the SL cycle, a fringing reef can transform into a barrier reef during a single episode of postglacial SL rise.

During subsequent SL cycles, the process is repeated: early postglacial fringing reefs retreat upslope until they encounter the barrier reef-flat platform from the previous cycle. Barrier-reef development then resumes, and a new reefal cap fills the remaining accommodation space created by subsidence. Eventually, after several cycles, subsidence and terrestrial erosion combine to displace the volcanic island below the SL highstand position, transforming the barrier into an atoll (Fig. 5). Such interaction between retreating fringing reefs and former reef-flat platforms during multiple SL cycles on gradually subsiding islands, therefore supports Darwin's 175 year-old claim of a genetic link between reef types.

### Exceptions and shelf-edge reefs

**Figure 1:**
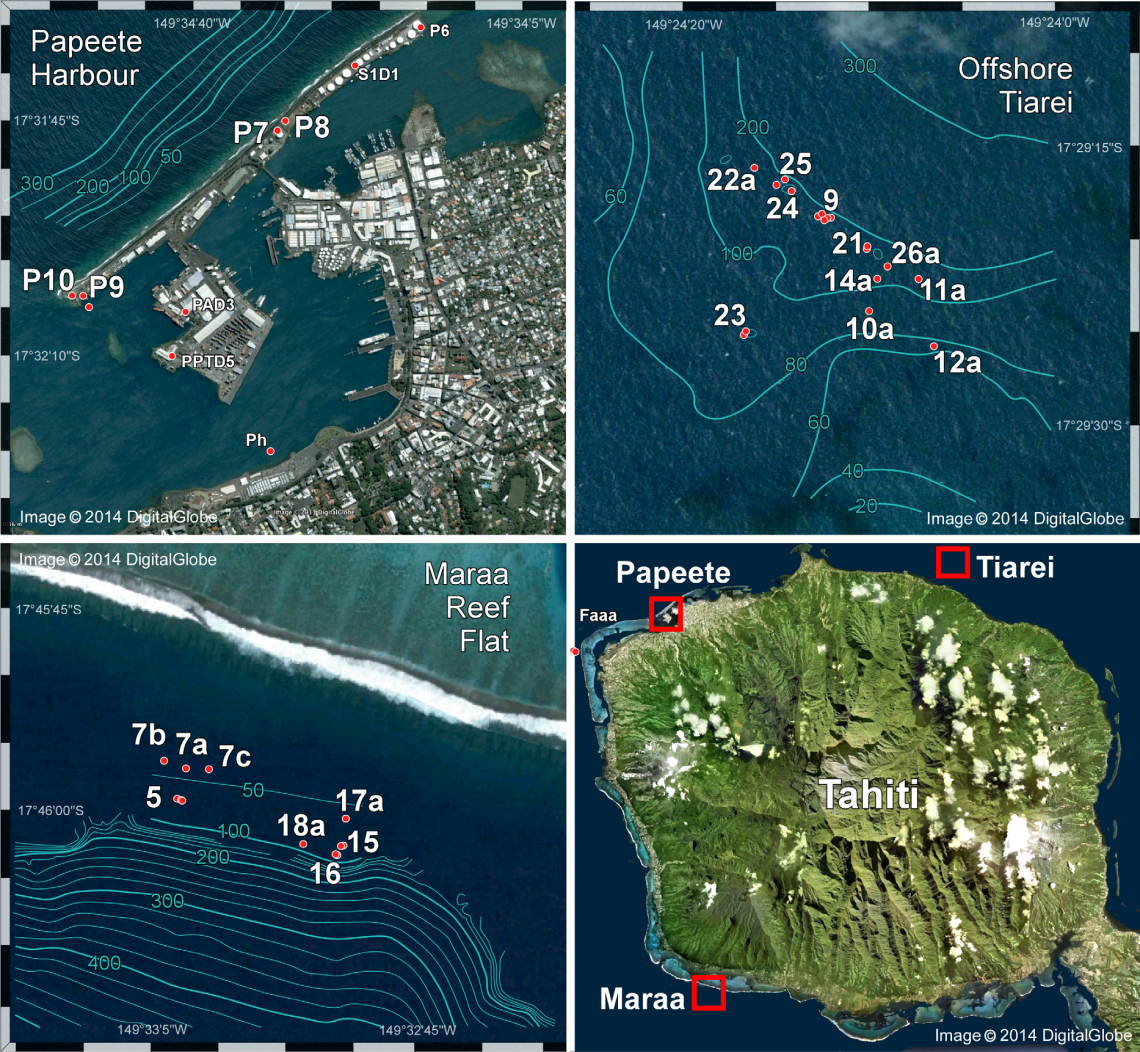
Digital Globe images of Tahiti, showing modern barrier and fringing reefs, IODP-310 drill locations on reef-front slope at Maraa and Tiarei, and previous drill sites at Papeete barrier reef and lagoon (Image credit: Modified and republished with permission from Digital Globe). Maraa and Tiarei bathymetry and hole-location data from ref. 20, Papeete data from ref. 19.

**Figure 2:**
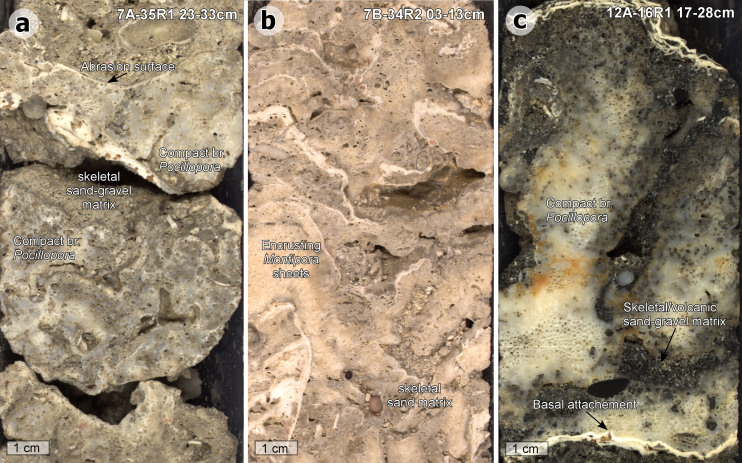
Surf-adapted corals of the fringing-reef unit at Maraa and Tiarei. (a), Small, bioeroded colonies of compact-branching *Pocillopora* sp. in a skeletal sand-gravel matrix (Maara). (b), Dense biofabric of encrusting sheets of *Montipora* sp. in skeletal sand-gravel matrix (Maara). (c), Compact branching colony of *Pocillopora verrucosa* showing bioerosion by clionid sponges, and skeletal/volcanic sand-gravel matrix (Tiarei).

In addition to linking the main reef types, our fringing-reef retreat model also highlights several factors that limit transitions between reef types. The first is illustrated by transition between barrier- and fringing-reef types on the west coast of Tahiti (Fig. 1). Local transitions such as these imply that variation may exist in the width or presence of the underlying reef-flat-platform, and where it is narrow or absent, only fringing reefs can form. Variable widths of reef-flats on modern fringing reefs commonly relate to differences in island slopes, which control the amount of reef progradation that can occur during interglacials^35^. This slope explanation is consistent with reef development on Tahiti, where the modern fringing reefs are built on the original steep volcanic slopes of the island, and barrier reefs on lower slopes affected by flank collapse^36^.

A second limitation may stem from the biogeographic distribution of corals, particularly prolific reef-builders such as *Acropora*. Census data show that diverse acroporid assemblages are present in island chains where Darwin's reef types are developed, such as the Society Islands, but rare or absent from those with poor or incomplete development of reef types^37^. In the Hawaiian-Emperor chain, for example, scarcity or lack of acroporids^38^ likely reduces the potential of reefs to accrete vertically during postglacial SL rise. This reduction may not only explain the lack of barrier reefs in the chain, but also the disproportionate number of submerged banks with drowned reefs^39^,^40^. The incomplete sequence of reef types in the Hawaiian chain has been recently attributed to it falling outside a narrow ‘Goldilocks zone’ where island subsidence and accretion rates are ‘just right’ for transitions between reef types^41^. While our results support the concept of a subsidence threshold for barrier-reef development (eg. ~1.2 mm yr^−1^ for the last cycle), it is worth noting that many island chains, including the Hawaiian chain, have a wide range of subsidence rates which encompass such thresholds, implying that a lack of acroporids and the resulting reduced accretion potential is more important in preventing the transition between fringing- and barrier-reefs.

In addition to these absences, the fringing-reef retreat model may also help explain reef development on the edges of more stable continental shelves. For example, highstand fringing reefs likely developed platforms close to shelf edges during mid-Pleistocene interglacials, when the amplitude of the SL cycle was reduced^42^. The development of platforms in this lower position would subsequently anchor postglacial fringing-reef retreat and produce barrier reefs that were repeatedly re-occupied during later interglacials, when higher-amplitude cycles dominated. Such re-occupation is clearly seen in core sequences from well studied shelf-edge barrier reefs^43^,^44^. Cores recovered from the reef flat of Ribbon Reef 5, in the northern Great Barrier Reef, consist of 5 shallow interglacial reef units as old as ~450 ka with identical coral and coralline algal assemblages, implying that the reef crest was reoccupied by later crests^43^,^45^. Similarly, cores from Kendec and Tenia islets on the barrier reef off New Caledonia, consist of 4 superimposed interglacial reef-flat units as old as ~400 ka^44^. Importantly, both the GBR and New Caledonia barrier-reefs only became fully established after the mid-Pleistocene (mid Brunhes) when high-amplitude SL cycles became dominant^44^,^45^,^46^.

While Darwin stressed the simple up-growth of fringing reefs fixed in position during relative SL change, data from both oceanic islands such as Tahiti and continental shelves like the GBR imply that fringing reefs are not fixed, but are inherently mobile systems forced to retreat and advance during transgressive and regressive stages of glacio-eustatic SL cycles. The anchoring of these mobile reefs by interglacial reef-flat platforms liberates them from coastal sediment flux thereby allowing acroporids to restore their vertical accretion potential and initiate the transition into a barrier reef. This process is optimal on young oceanic islands because thermotectonic subsidence lowers platforms so that they anchor retreating fringing reefs early during postglacial SL rise, giving rise to barrier reefs within a single SL cycle (Fig. 5).

Darwin's intuition about the genetic link between reef types was right, but his mechanism of vertical growth during subsidence is untenable under glacio-eustasy. The transition between reef types, as finally revealed at Tahiti, stems from a multicyclic interaction between postglacial SL rise, fringing-reef retreat, and former interglacial reef-flat platforms. This interaction, and the transition it causes between reef types, can be prevented by the absence of important reef-building corals, or by excessive rates of subsidence or SL rise. Thus our model not only provides an explanation of why fringing reefs transform into barrier reefs then atolls, but also why this transition is absent in certain island chains, and why shelf-edge barriers and other reefs were repeatedly re-occupied during glacio-eustatic SL cycles.

## Methods

### Sedimentary core analysis

**Figure 3:**
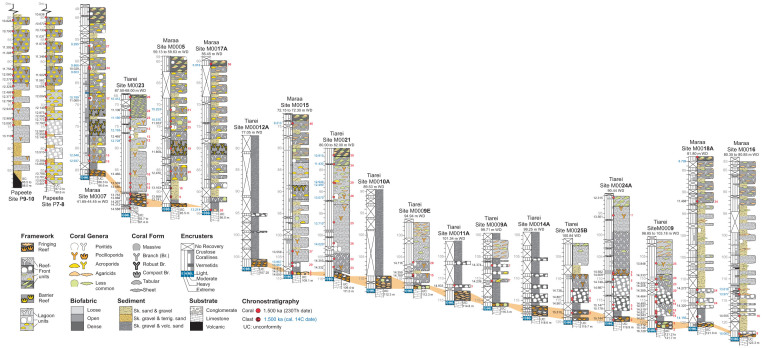
Site summary profiles showing sedimentary units, coral assemblages, ages, and paleowater depths (in red) for Maraa, Tiarei, and Papeete. Excluding Papeete, and Maraa site 17A, each hole has a thin basal fringing-reef unit overlain by framework units in which the paleowater depths increase up-core. The basal fringing-reef unit overlaps in elevation between sites 16 and 12A, and between sites 7 and 5, showing that it forms two layers (highlighted in orange). The break between layers at sites 12A and 5 coincides with Meltwater Pulse 1a.

The archive core-half of each hole was logged and scanned in the IODP Gulf Coast Repository at TAMU in College Station, Texas in November 2010. Core logging followed a sedimentary Reef Core Analysis Protocol (Supplementary Fig. S5 online), which differentiates skeletal-framework units composed predominantly of mutually-supported in-situ corals, from detrital units composed predominantly of grain-supported clasts. That differentiation is based on the identification of in-situ corals, which can be problematic in narrow diameter cores^50^. We used combinations of presence/absence indicators including, in order of confidence, basal-attachment surfaces, coral orientation, and consistent upward-oriented geopetals >0.3 cm diameter which contained lithified sediment^51^. We found these criteria to be generally reliable in massive and encrusting colonies where basal attachment surfaces are common, but not in branching and platy colonies where basal attachment surfaces are rare and orientation can be deceptive (especially on sloping substrates). In these cases, we found that consistency in orientation and/or mutual proximity of coral colonies was the key to a reliable differentiation of framework from detrital units. We combined the sedimentological analysis of framework/detrital units with the taxonomy of the coralgal assemblages using data from ref. 21. Distinguishing features, elevation and continuity of units were confirmed by comparing closely-spaced holes from each site (except for sites where only single holes were drilled). This allowed us to produce a summary log representative of unit character and elevation at each site (Fig. 3). Units and their boundary elevations were also checked, and the assumed default position of the core at the top of the coring run was adjusted in some cases based on facies continuity and downhole imagery. Elevations of dated samples from such adjusted core positions were changed accordingly. None of the elevations of unit boundaries were corrected for island subsidence (Supplementary Discussion).

### Dating

We used ^230^Th ages reported by ref. 17, and measured ^14^C ages reported by refs. 21, 22, 47 and 48. We corrected the measured ^14^C ages for the ocean reservoir effect, and then calibrated the ages to calendar (Cal) years using the curve reported in ref. 49, which allows input of different reservoir ages. Due to growing evidence of temporal variation in the ocean reservoir age^52^,^53^,^54^, we attempted to hindcast the reservoir age of Tahiti surface waters using paired ^14^C and ^230^Th ages reported by ref 69. This calculation stems from the difference between ^14^C ages calibrated in ref. 49, and their respective absolute ages, determined by ^230^Th. Corals growing during the Late Glacial (>14.5 ka) and Younger Dryas (11.2 to 12.9 ka) have significantly reduced reservoir values of 280 years, whereas warmer Bolling/Allerod and Holocene stages have modern reservoir values of 400 years or greater^55^. As a consequence we corrected the reported ^14^C ages using these two reservoir variations (Fig. 3). In light of this temporal variation in reservoir age, we consider calibrated ^14^C ages to have a higher level of uncertainty than their analytical error and assign them less confidence than the ^230^Th data.

### Paleowater depth

To reconstruct the paleowater depth and check interpretations made from coralgal assemblages^21^, we compared age/elevation data from framework units in each core with the highest coeval corals in all cores, including the Papeete barrier-reef cores^16^. Subtracting the elevation of the highest coeval coral gives a minimum paleowater depth, if it is assumed that the highest coral grew at SL (Supplementary Fig. S6). If the highest coeval coral is from an assemblage with a distinctive depth range however, the depth of the assemblage base can be used to give maximum paleowater depth. Hence for each dated sample from the Tiarei and Maraa cores, a minimum and maximum paleowater depth can be calculated, and an average of the two will give the most objective value. A similar approach is to reconstruct a minimum SL curve from all coral age/elevation data and then compare each coral in each framework unit with that reconstruction (Supplementary Fig. S6). Age and elevation data from the Papeete barrier-reef cores are particularly useful for establishing paleowater depth of the Tiarei and Maraa framework units, given that they sampled shallow reef-front, crest, and back-reef units that developed in waters <8 m deep during the last 14 ka^16^. For paleowater depth reconstruction of units older than 14 ka, we plotted all age/elevation data and compared coeval corals in the same way, using coral morphology and suites of secondary encrusters to identify the shallowest surf-zone facies.

### Reef Accretion

**Figure 4:**
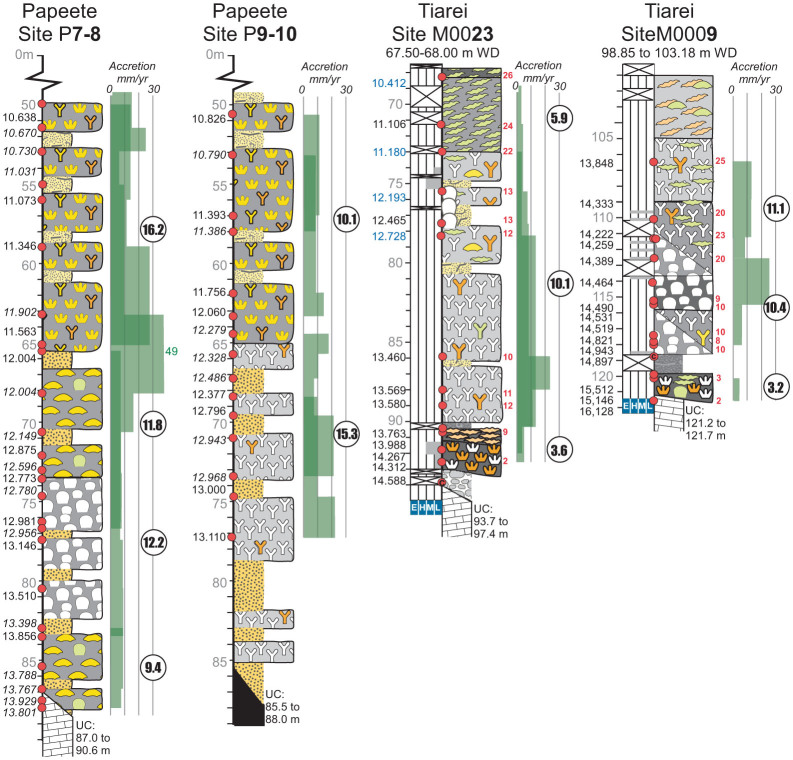
Accretion rates of fringing-reef and other framework units from Tiarei and Papeete. Transient accretion occurs in the interval between dated samples and shows the potential of the reef to accrete vertically over a limited time. Green shading in the accretion column shows transient rates calculated for different holes at the same site (dark green shows overlap in transient accretion between holes). The average accretion within each unit is shown by circled values. The reef-crest unit at Papeete, for example, shows transient accretion of up to 49 mm yr^−1^, but has an average accretion of ~16 mm yr^−1^ between 65 and 50 m in P7-8. By contrast, the fringing-reef unit shows transient accretion of up to 5 mm yr^−1^ but averages only ~3.5 mm yr^−1^ between holes. Ages in italics distinguish data from different holes. Ages in blue are calendar age calibrations of ^14^C ages (Methods). Closely spaced holes from site 9 (9b,c and d) are combined to calculate accretion rates.

**Figure 5:**
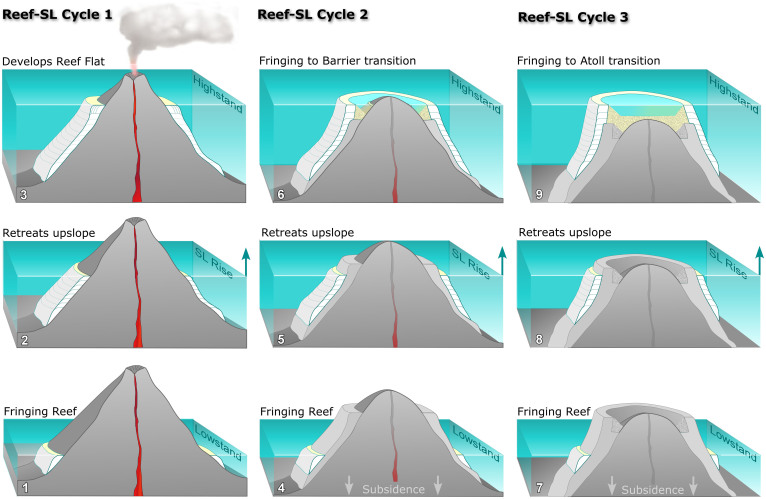
Fringing-reef retreat controls the transition between reef types during glacio-eustatic SL cycles. The first SL cycle starts with a fringing reef during the glacial lowstand (1). During the rapid postglacial transgression, the reef is forced to retreat upslope due to its low accretion rate (2). Only as SL rise slows into the interglacial, can the fringing reef advance seawards producing a reef flat (3). Following glacial SL fall, the second cycle begins, as before, with a fringing reef retreating upslope with the transgression (4). This time, however, it encounters the former reef-flat platform at a lower elevation due to island subsidence. The slight reverse slope of the platform prevents further upslope retreat and fixes the reef on its rim, producing a lagoon that traps coastal sediment (5). Isolated from sediment, the reef is colonised by fast-growing acroporids which allow it to accrete vertically and keep pace with SL rise, producing a barrier reef (6). In the final cycle, subsidence and erosion displace the volcanic peak below the highstand elevation (7), so that when the fringing reef reaches the rim of the former barrier-reef flat (8), it can accrete vertically and transform into an atoll (9).

For each continuous framework sequence in each hole, we measured vertical accretion between consecutive ages, where these ages are from different corals >1 m apart, with an age difference greater than the analytical dating error (~50 years). Value differences between consecutive ages provides a measure of transient accretion, which can be used to determine the vertical accretion potential of a unit. Where more than one transient-accretion value is available from each unit, they are combined to provide an average accretion rate for that unit. Transient values are plotted on the core logs in Fig. 4 so that changes in accretion rate can be identified within and between framework units.

## Author Contributions

P.B. initiated the study, collected and analysed the data with M.G.-C. and wrote the manuscript. All authors (P.B., M.G.-C., E.A., J.C.B., C.B., D.M.K., T.S., J.M.W., C.D.W.) contributed to the editing of the manuscript.

## Supplementary Material

Supplementary Information Postglacial Fringing-Reef to Barrier-Reef conversion on Tahiti links Darwin's reef types

## References

[b1] Darwin C. R. The structure and distribution of coral reefs. Being the First Part of the Geology of the Voyage of the Beagle, Under the Command of Capt. Fitzroy, R.N. During the Years 1832 to 1836. (Smith Elder, London1842).

[b2] Penck A. Schwankungen des Meeresspiegels. Geographica. Gesellschaft. München 7, 47–116 (1882).

[b3] Daly R. The glacial-control theory of coral reefs. Proc. Amer. Acad. Arts Sci. 51, 155–251 (1915).

[b4] Schlanger S. O. Subsurface geology of Eniwetok Atoll. U.S.G.S. Prof. Paper P 0260-BB, 991–1066 (1963).

[b5] Szabo B. J., Tracey J. I. & Goter E. R. Ages of subsurface stratigraphic intervals in the Quaternary of Enewetak Atoll, Marshall Islands. Quat. Res. 23, 54–61 (1985).

[b6] Gray S. G., Hein J. R., Hausmann R. & Radtke U. Geochronology and subsurface stratigraphy of Pukapuka and Rakahanga atolls, Cook islands: late quaternary reef growth and sea level history. Palaeogeogr. Palaeoclimatol. Palaeoecol. 91, 377–394 (1992).

[b7] Lincoln J. M. & Schlanger S. O. Atoll stratigraphy as a record of sea level change: problems and prospects. J. Geophys. Res. 96, 6727–6752 (1991).

[b8] Scott G. A. J. & Rotondo G. M. A model to explain the differences between Pacific plate island-atoll types. Coral Reefs 1, 139–150 (1983).

[b9] Ladd H. S., Ingerson E., Townsend R. C., Russell M. & Stephenson H. K. Deep drilling on Eniwetok Atoll, Marshall Islands. Bull. Amer. Assoc. Petrol. Geol. 37, 2257–2280 (1953).

[b10] Vaughan T. W. Fossil corals from Central America, Cuba, and Porto Rico, with an account of the American Tertiary, Pleistocene, and Recent coral reefs. US Nat. Mus. Bull. 103, 189–524 (1919).

[b11] Purdy E. G. Reef configurations: Cause and effect. In Laporte, L. F., ed., Reefs in time and space: Soc. Econ. Paleont. Mineral. Spec. Pub. 18, 9–76 (1974).

[b12] Purdy E. G. & Winterer E. L. Contradicting barrier reef relationships for Darwin's evolution of reef types:. Int. J. of Earth Sciences 95, 143–167 (2006).

[b13] Chappell J. Sea-Level Changes and Coral Reef Growth. In: Perspectives on Coral Reefs, Edited by Barnes, D.J. The Australian Institute of Marine Science, Manuka, A.C.T., pp. 46–55 (1983).

[b14] Stein M. *et al.* TIMS U-series dating and stable isotopes of the last interglacial event in Papua New Guinea. Geochim. Cosmochim. Acta 57, 2541–2554 (1993).

[b15] Steers J. A. & Stoddart D. R. [The origin of fringing reefs, barrier reefs and atolls]. Biology and Geology of Coral Reefs. Geology, vol. 2, [Jones, O. A., Endean, R. (Eds.)] [21–57] (Academic Press1977).

[b16] Bard E., Hamelin B. & Delanghe-Sabatier D. Deglacial Meltwater Pulse 1B and Younger Dryas sea levels revisited with boreholes at Tahiti. Science 327, 1235–1237 (2010). 20075212 10.1126/science.1180557

[b17] Deschamps P. *et al.* Ice-sheet collapse and sea-level rise at the Bølling warming 14,600 years ago. Nature 483, 559–564 (2012). 22460900 10.1038/nature10902

[b18] Montaggioni L. F. *et al.* Continuous record of reef growth over the past 14 k.y. on the mid-Pacific island of Tahiti. Geology 25, 555–558 (1997).

[b19] Cabioch G., Camoin G. F. & Montaggioni L. F. Postglacial growth history of a French Polynesian barrier reef tract, Tahiti, central Pacific. Sedimentology 46, 985–1000 (1999).

[b20] Camoin G. F., Iryu Y. & McInroy D. Expedition 310 scientists. Proc. IODP, 310 (2007). College Station TX (Integrated Ocean Drilling Program Management International, Inc.). 10.2204/iodp.proc.310.101 (2007).

[b21] Abbey E. *et al.* Variation in deglacial coralgal assemblages and their paleoenvironmental significance: IODP Expedition 310, “Tahiti Sea Level”. Global Planet. Change 76, 1–15 (2011).

[b22] Camoin G. F. *et al.* Reef response to sea-level and environmental changes during the last deglaciation: Integrated Ocean Drilling Program Expedition 310, Tahiti Sea Level. Geology 40, 643–646 (2012).

[b23] Kaandorp J. A. Morphological analysis of growth forms of branching marine sessile organisms along environmental gradients. Mar. Biol. 134, 295–306 (1999).

[b24] Madin J. S. Mechanical limitations of reef corals during hydrodynamic disturbances. Coral Reefs 24, 630–635 (2005).

[b25] Sebens K. P. & Done T. J. Water flow, growth form and distribution of scleractinian corals: Davies Reef (GBR), Australia. Proc. 7th Int. Coral Reef Symp., Guam, 1992, vol 1, 557–568 (1994).

[b26] Madin J. S. & Connolly S. R. Ecological consequences of major hydrodynamic disturbances on coral reefs. Nature 444, 477–480 (2006). 17122855 10.1038/nature05328

[b27] Laurent V. K., Maamaatuaiahutapu J., Maiau A. & Varney P. Atlas climatologique de la Polynesie francaise. (MeteoFrance, Papeete, 2004).

[b28] Rodríguez-Martínez R. E., Jordán-Garza A. G., Maldonado M. A. & Blanchon P. Controls on coral-ground development along the Northern Mesoamerican Reef Tract. PLoS ONE 6(12): e28461 10.1371/journal.pone.0028461 (2011). 22194839 10.1371/journal.pone.0028461 PMC3237443

[b29] Hongo C. & Kayanne H. Key species of hermatypic coral for reef formation in the northwest Pacific during Holocene sea-level change. Mar. Geol. 279, 162–177 (2011).

[b30] Kojis B. L. & Quinn N. J. Seasonal and depth variation in fecundity of Acropora palifera at two reefs in Papua New Guinea. Coral Reefs 3, 165–172 (1984).

[b31] Fabricius K. & De'ath G. Environmental factors associated with the spatial distribution of crustose coralline algae on the Great Barrier Reef. Coral Reefs 19, 303–309 (2001).

[b32] Thomas A. L. *et al.* Penultimate deglacial sea-level timing from uranium/thorium dating of Tahitian corals. Science 324, 1186–1189 (2009). 19390000 10.1126/science.1168754

[b33] Ménabréaz L., Thouveny N., Camoin G. & Lund S. P. Paleomagnetic record of the late Pleistocene reef sequence of Tahiti (French Polynesia): a contribution to the chronology of the deposits. Earth Planet. Sci. Lett. 294, 58–68 (2010).

[b34] Kan H., Hori N., Kawana N., Kaigara T. & Ichikawa K. The evolution of a Holocene fringing reef and island: reefal environmental sequence and sea level change in Tonaki Island, the Central Ryukyus. Atoll Res. Bull. 443, 1–20 (1997).

[b35] Kennedy D. M. & Woodroffe C. D. Fringing reef growth and morphology: a review. Earth-Sci. Rev. 57, 255–277 (2002).

[b36] Hildenbrand A., Gillot P. & Marlin C. Geomorphological study of long-term erosion on a tropical volcanic ocean island: Tahiti-Nui (French Polynesia). Geomorphology 93, 460–481 (2008).

[b37] Wallace C. Staghorn corals of the world. (CSIRO Publishing, Collingwood, 1999).

[b38] Grigg R. W. Community structure, succession and development of coral reefs in Hawaii. Mar. Ecol. Prog. Ser. 11, 1–14 (1983).

[b39] Grigg R. W. *et al.* Drowned reefs and antecedent karst topography, Au'au Channel, SE Hawaiian Islands. Coral Reefs 21, 73–82 (2002).

[b40] Webster J. M. *et al.* Drowning of the -150 m reef off Hawaii: a casualty of global meltwater pulse 1A? Geology 32, 249–252 (2004).

[b41] Toomey M., Ashton A. D. & Perron J. T. Profiles of ocean island coral reefs controlled by sea-level history and carbonate accumulation rates. Geology 41, 731–734 (2013).

[b42] Miller K. *et al.* The Phanerozoic record of global sea-level change. Science 310, 1293–1298 (2005). 16311326 10.1126/science.1116412

[b43] Webster J. M. & Davies P. J. Coral variation in two deep drill cores: significance for the Pleistocene development of the Great Barrier Reef. Sedim. Geol. 159, 61–80 (2003).

[b44] Cabioch G. *et al.* The chronology and structure of the western New Caledonian barrier reef tracts. Palaeogeogr. Palaeoclimatol. Palaeoecol. 268, 91–105 (2008).

[b45] Braithwaite C. J. R. *et al.* The Great Barrier Reef: the chronological record from a new borehole. J. Sediment. Res. 74, 298–310 (2004).

[b46] Alexander I. *et al.* New constraints on the origin of the Australian Great Barrier Reef: results from an international project of deep coring. Geology 29, 483–486 (2001).

[b47] Heindel K., Wisshak M. & Westphal H. Microbioerosion in Tahitian reefs: a record of environmental change during the last deglacial sea-level rise (IODP 310). Lethaia 42, 322–340 (2009).

[b48] Inoue M. *et al.* Trace element variations in fossil corals from Tahiti collected by IODP Expedition 310: reconstruction of marine environments during the last deglaciation (15 to 9 ka). Mar. Geol. 271, 303–306 (2010).

[b49] Fairbanks R. G. *et al.* Radiocarbon calibration curve spanning 0 to 50,000 years BP based on paired 230Th/234U/238U and 14C dates on pristine corals. Quat. Sci. Rev. 24, 1781–1796 (2005).

[b50] Blanchon P. Continuous record of reef growth over the past 14 ky on the mid-Pacific island of Tahiti: Comment and Reply. Geology 26, 479 (1998).

[b51] Blanchon P. & Perry C. T. Taphonomic differentiation of Acropora palmata facies in cores from Campeche Bank Reefs, Gulf of Mexico. Sedimentology 51, 53–76 (2004).

[b52] Kromer B. *et al.* Late Glacial 14C ages from a floating, 1382-ring pine chronology. Radiocarbon 46, 1203–1209 (2004).

[b53] Muscheler R. *et al.* Tree rings and ice cores reveal C-14 calibration uncertainties during the Younger Dryas. Nature Geosci. 1, 263–267 (2008).

[b54] Singarayer J. S. *et al.* An oceanic origin for increase of atmospheric radiocarbon during the Younger Dryas,. Geophys. Res. Lett. 35, L14707 (2008).

[b55] Paterne M. *et al.* Paired 14C and 230Th/U dating of surface corals from the Marquesas and Vanuatu (sub-equatorial Pacific) in the 3000 to 15,000 cal yr interval. Radiocarbon 46, 551–566 (2004).

