# Supplementary material for: Postglacial Fringing-Reef to Barrier-Reef conversion on Tahiti links Darwin's reef types
Source: Sci Rep. 2014 May 21;4:4997. doi: 10.1038/srep04997 (PMC4028904; doi:10.1038/srep04997)
Supplement: Supplementary Information — Postglacial Fringing-Reef to Barrier-Reef conversion on Tahiti links Darwin's reef types [file srep04997-s1.pdf]

Supplementary Information

Postglacial Fringing-Reef to Barrier-Reef conversion on Tahiti links Darwin's reef types

Paul Blanchon*, Marian Granados-Corea, Elizabeth Abbey, Juan C. Braga, Colin Braithwaite, David M. Kennedy, Tom Spencer, Jody M. Webster and Colin D. Woodroffe. *blanchons@gmail.com

Contents: Supplementary Figures S1-6; Supplementary Discussion with references.

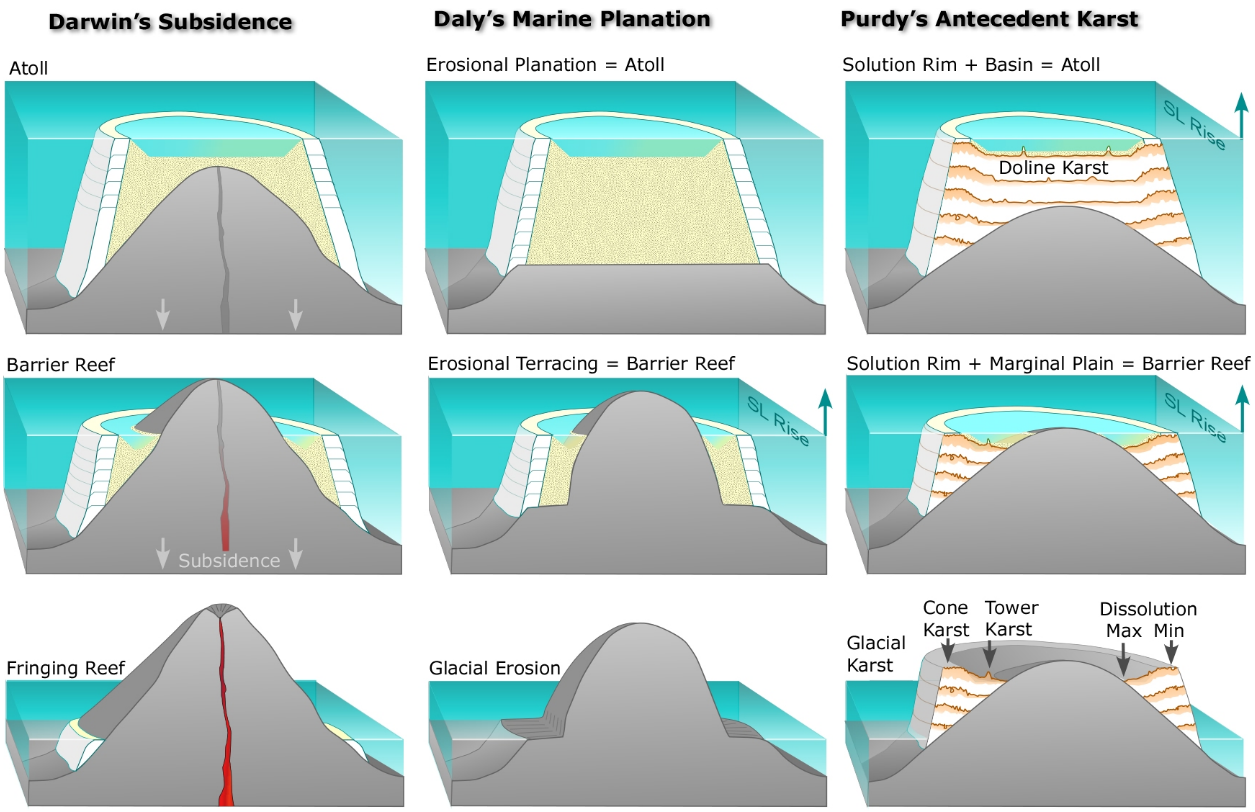

Supplementary Figure S1 | Historical progression of ideas explaining fringing, barrier and atoll reef types. Darwin's subsidence theory[1] suggests reef types are genetically related and form during a single episode of relative SL rise produced by progressive island subsidence. Daly's marine planation theory[3] suggests they are unrelated, but result from differential wave erosion of unprotected shores during glacially lowered SL, with barrier reefs forming on terraces, and atolls on wave-levelled platforms during postglacial SL rise. Purdy's antecedent karst theory[11] also suggests reef-types are unrelated, and result from subaerial dissolution during lowstands, with rims undergoing less dissolution than platform interiors leading to shallow atoll-like basins on carbonate platforms and barrier-like half basins on continental or island margins. It posits that glacial karst foundations control the morphology of modern reefs.

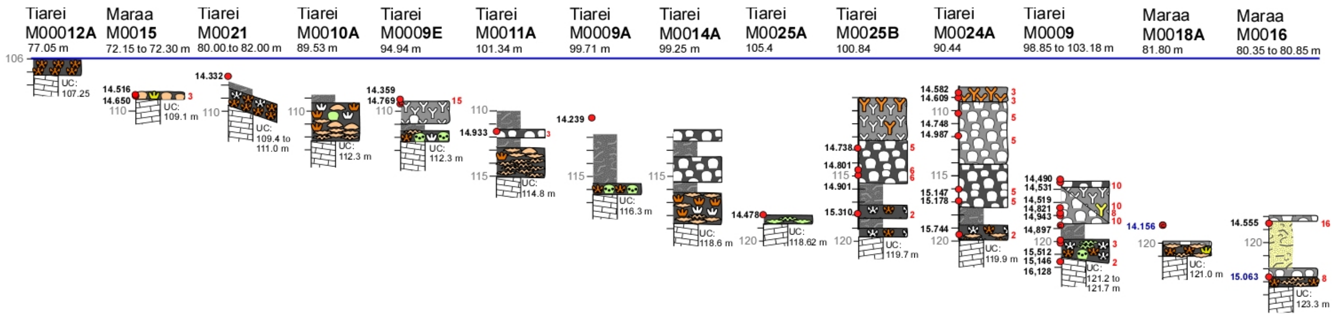

Supplementary Figure S2 | Fringing reef and reef-front development between 16.1 to 14.5 ka (ie. prior to Meltwater pulse 1a). Shallow reef development is largely absent at Maraa sites, and patchy at Tiarei sites, with best patch development at sites 25B and 24A, which are drilled on the same reef-front pinnacle. Note absence of patch in site 25A which was drilled 5 m from 25B, illustrating discontinuous nature of reef-front development at Tiarei. Legend same as Fig. 3.

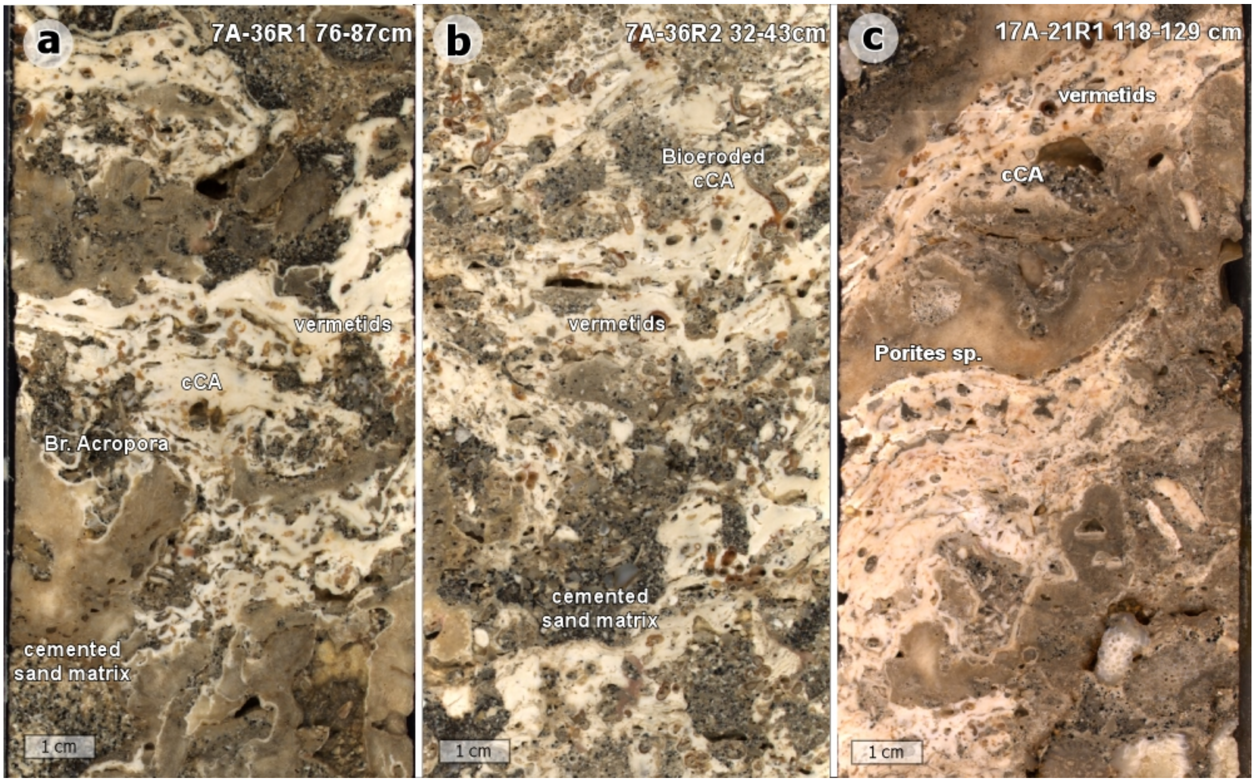

Supplementary Figure S3 | Pleistocene reef-flat platform unit, showing intergrown crustose-coralline algae, vermetids and stubby branched corals, which are typical of reef-flat settings. **a,** and **b,** from a 3 m-thick unit at site 7A, show how intergrown fabric has been moderately bioeroded and the interstices filled with a cemented well-sorted medium-sized skeletal/volcanic sand. **c,** from a 2.5 m unit at site 17A, shows a similar intergrown fabric, but with thicker, less bioeroded corallines within a unit dominated by larger corals, which is more typical of shallow reef-front settings (such as spur and groove).

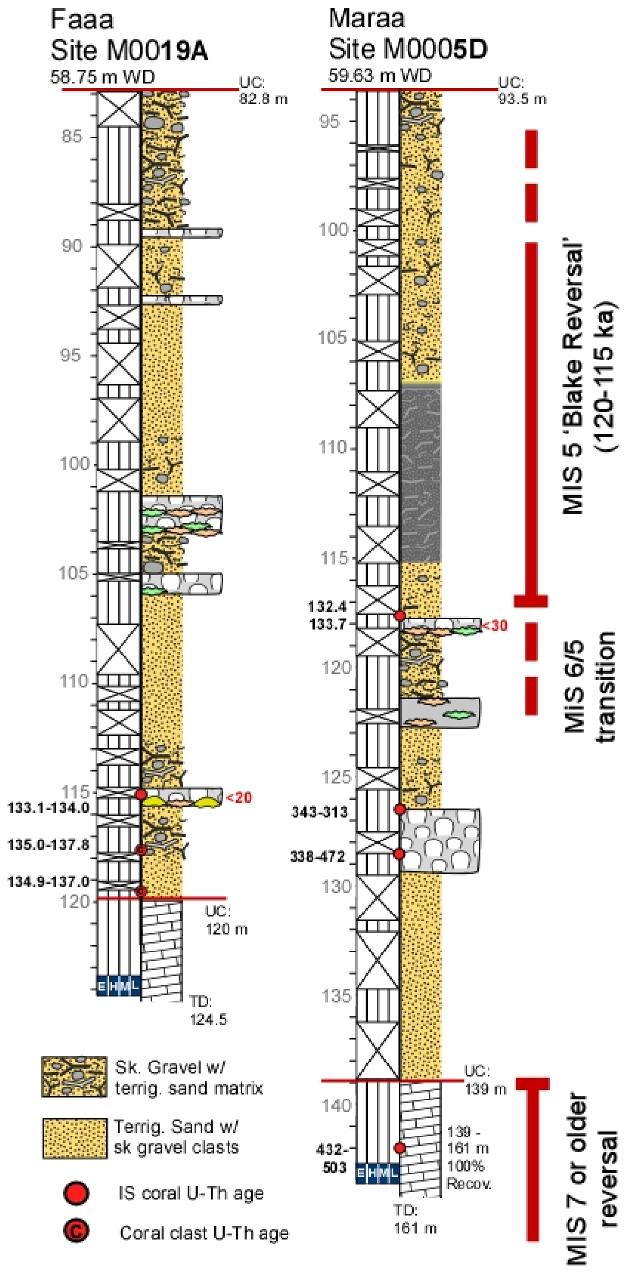

Supplementary Figure S4 | Composition, ^{230}Th age, and magnetostratigraphy of reef-flat platform which underlies the highest postglacial fringing- reef unit and the basal barrier-reef unit. Upper section consists of poorly consolidated (and recovered) detrital unit with isolated patches of coral framework that return Last Interglacial ages, and has a magnetostratigarphic signature consistent with the Blake Event between 120-115 ka[33]. This is underlain by a well-consolidated unit of uncertain age due to large true-age variation in measured ages.

Reef Core Analysis Protocol

Biofabric

Element support and packing[9]
1. ***Dense***: colony supported, tightly packed with little or no intercolony space, line contacts common.
2. ***Open***: colony supported, closely packed with easily distinguishable intercolony space, line contacts rare, point contacts common.
3. ***Loose***: colony supported, but loosely packed and dominated by intercolony space, with rare point contacts.
4. ***Dispersed***: not colony supported, with colony spacing <1 m or 2 unit distance.
5. ***Sparse***: not colony supported, colony spacing >1 m or 2 unit distance.

Element shape-uniformity[10]
1. ***Monomorphic***: biofabric dominated by single colony shape type.
2. ***Bimorphic***: biofabric dominated by two shape types.
3. ***Multimorphic***: biofabric dominated by >2 shape types.

Unit Geometry
1. ***Layer***: units have bedded geometry w/ sub-horizontal upper & lower surfaces.
2. ***Mosaic***: juxtaposed group of units each with discontinuous horizontal or vertical extent.
3. ***Patch/Pinnacle***: unit isolated within another (Pinnacle >5 m height).
4. ***Lens***: unit isolated within another but with limited vertical extent.

Unit Contacts
1. Sharp
2. Erosive (evidence of truncation)
3. Transitional
4. Intercalated
5. Inferred

Framework Plot [12]

On a 3-component triangular diagram, plot normalized proportion of:
- **F**: framework (primary + secondary framework)
- **M**: interstitial matrix, infauna, cavities (incl. cavity fill cement)
- **D**: Bioclastic detritus (ie. coral gravel fragments).

Reef Framework

Primary framework
1. Main Reef-Building Corals[1]

Secondary framewok
1. Corallines: crustose (cCA), branched (bCA).
2. Encrusters: Vermetids, Foraminifera[4], Serpulids.
3. Biolithite and its biofabric
4. Marine cement varieties[5]

Coral In-situ Indicators:
1. Encrusting basal contact present
2. Up-oriented Coral or corallites
3. Colonies lack evidence of fragmentation.
4. Consistent up-oriented geopetals >0.3 cm diameter with lithified sediment fill.
5. Consistency in orientation or mutual proximity of colonies.

Coral shape type and size[2]
1. ***Massive***: (>10 cm vertical)
   small: 10-25 cm
   medium: 25-50 cm
   large: 50-150 cm
   super: >150 cm
2. ***Branch***: (br.)
   Br. length: long >10 cm, short 1-10 cm
   Br. thickness: delicate <1 cm, thick 1-2 cm, robust >2 cm.
   Compact Br: short, robust with large basal attachment.
3. ***Plate*** *(>1, <10 cm vertical),*
   Pt, (thin 1-5 cm), **PT**, (thick 5-10 cm),
   Pc (compound), **Pe** (encrusting).
4. ***Sheet*** *(< 1cm)*:
   Sc (compound), **Se** (encrusting).

Reef Detritus

Bioclasts (gravel-size fragments)
composition, size (Wentworth scale[6]) visual sorting (size and/or shape), texture[7], fabric.

Indicators of transport:
1. Inclined or inconsistent colony orientation
2. Colonies fragmented or abraded.
3. Inclined or inconsistent geopetal orientation
4. Poor colony condition (bioeroded, multiple encrusters)

Matrix (sand, silt, clay-size grains) Size, sorting, texture (8), fabric

Condition

Skeletal Preservation State[11]
1. ***Fresh*** skeletal features (features unmodified)
2. ***Modified*** skeletal features (species identification still possible)
3. ***Semi-obscurred*** skeletal features (species id possible, but unreliable)
4. ***Obscurred*** skeletal features (no species id possible)

Bioerosion Ichnotaxa[3]
Entobia
Gastrochaenolites
Trypanites

Bioerosion Intensity
Small galleries
1. *light* (<10% host removal)
2. *moderate* (10-30% removal)
3. *heavy* (>30 % removal)

Large Galleries
1. light (<30 removal)
2. moderate (30-60 removal)
3. heavy (>60 removal)

Encrustation Intensity
1. *light* (<0.5 cm thick)
2. *moderate* (0.5-1.5 cm thick)
3. *heavy* (1.5-3.0 cm thick)
4. extreme (>3.0 cm thick)

Diagenetic State
1. ***Unaltered*** (original mineral phase intact)
2. ***Cemented*** (primary pore occlusion)
3. ***Leached*** (modification of primary or generation of secondary pores)
4. ***Recrystallized*** (inversion or replacement of original mineral phase)

Framework Name

±C (B+P+S) framestone where:
B is colony biofabric packing
P is primary framework taxa
S is colony shape
C is a modifier based on condition, detrital elements, shape uniformity, etc.

Eg: heavily bioeroded, loose, Acropora branch framestone

Supplementary Figure S5 | Core description protocol used for reefal sedimentary units (Modified from ref 56). 1. Following ref 57. 2. Modified from ref 56. 3. Following ref 58-60. 4. Following ref 61. 5. Following ref 62. 6. Modified by ref 63. 7. Following ref 64. 8. Following ref 65. 9. Modified from ref 66. 10. Modified from ref 67. 11. Modified from ref 68. 12. Modified from ref 69.

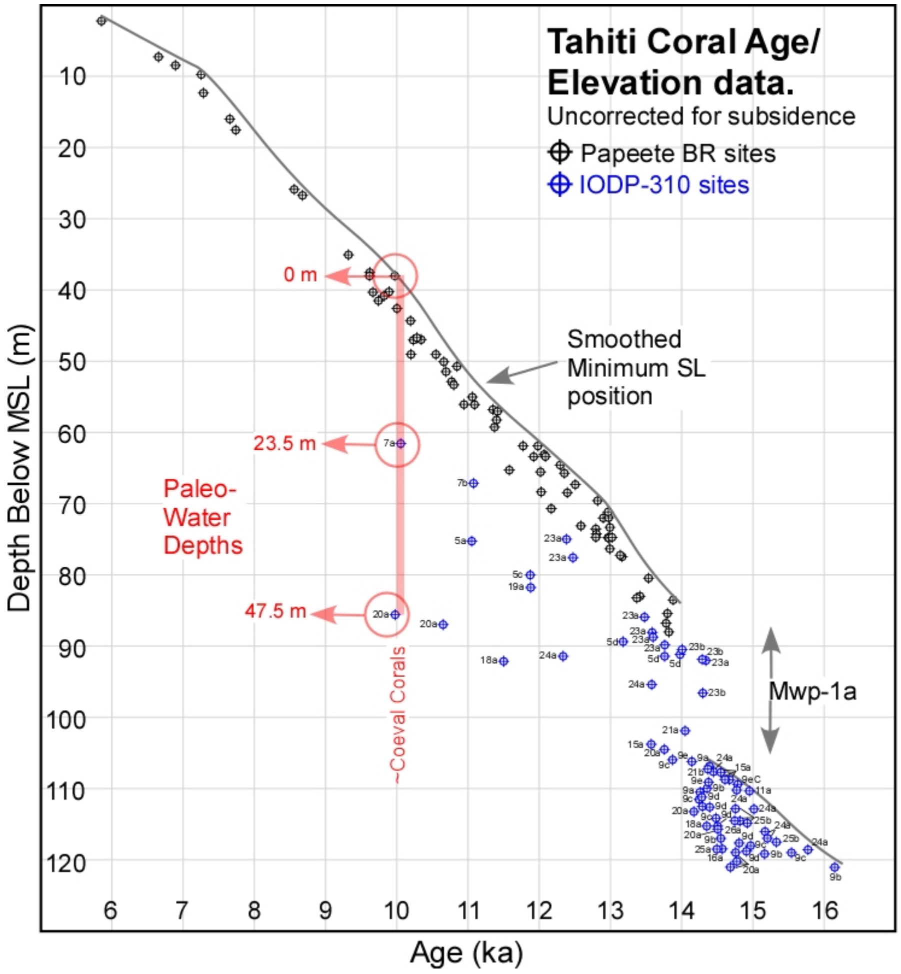

Supplementary Figure S6 | Paleowater depth reconstruction derived from elevation comparison between coeval corals, assuming that the highest coral is at, or close to mean minimum SL (defined as a smoothed curve fitted to the highest coral elevations). U-Th data from refs. 16 and 17.

Supplementary Discussion:

Age of the Pleistocene reef-flat platform and subsidence at Tahiti.

All holes drilled during the IODP Expedition-310 penetrated the underlying Pleistocene substrate but only three holes at Maraa and Tiarei recovered more than 10 m. The longest sequence at Maraa hole 5D was 86 m, followed by 25 m at 9D and 21.5 m at hole 8A. Two holes drilled at Faaa, just offshore of the Papeete barrier reef, also recovered 34-39 m of Pleistocene substrate (Fig. 1). In the holes at Maraa and Faaa, the Pleistocene sequence can be subdivided into an upper, poorly-consolidated unit with low recoveries, and a lower, well-consolidated unit with high recoveries (100% in some cases). Such differences imply that the contact between these units is an unconformity.

Radiometric dating of corals[32] in the upper poorly-consolidated unit at these two sites provides age constraints on the Pleistocene reef-flat platform that lies beneath the postglacial sequence at ~85 m (Supplementary Fig. S4). In Faaa hole 19A, replicate ^{230}Th ages of ~133-134 ka were measured in a *Porites* head at 115 m (~32 m below the platform surface at 83 m). Similarly, in Maraa hole 5D, replicate

ages of ~132-134 ka were measured in a *Porites* head at 117 m (~24 m below the platform surface at 93.5 m). These ages are considered to be reliable given that replicate ages only show true-age variations of ~2 ka[70]. Furthermore, a reversal in the paleomagnetic signature in this hole between 120-100 m has been attributed to the Blake Event that occurred between 120-115 ka during MIS-5e[33]. Combined, these data indicate that the upper poorly consolidated unit formed during the Last Interglacial (LIG). And although no age data exists for the 5-15 m sequence above 100 m depth, the sedimentary sequence in both holes is conformable over this interval, implying that the reef-flat platform formed during LIG (Supplementary Fig. S4).

A LIG age for the 85 m reef-flat platform would imply that the subsidence rate of Tahiti is greater than the 0.25 mm yr^{-1} estimate derived from a 550 ka basalt recovered in hole P7 in the Papeete barrier-reef[16]. Higher subsidence rates have been measured in ref. 71 which used several independent geodetic techniques and tide-gauge data to show an average rate of 0.5 mm yr^{-1} (but with large uncertainties). Furthermore, MIS 9 ages were obtained[72] from corals of the Maraa sequence in hole 5D between 126-128 mbsl suggesting a maximum constraint on subsidence for the past 325 ka of 0.39 ±0.03 mm yr^{-1}. However replicate ages measured over that 2 m interval have a true-age variation of 184 ka (n=12) for both closed and open systems, implying considerable uncertainty for the MIS-9 age determination and subsidence rates calculated from it. A similar magnitude of true-age variation was found from samples from the well-consolidated unit at the base of hole 5D, making it difficult to use any of these data to calculate subsidence rates.

As noted above, more reliable ages have been obtained from coral colonies that grew during the LIG in holes 5D and 19A (Supplementary Fig. S4). In hole 5D these corals had replicate ^{230}Th ages from 132-138 ka, and in 19A replicate ages from 133-137 ka[32]. In addition, ages in 5D were adjusted for open-system diagenetic alteration and returned corrected ages of 128-136 ka[32]. Within this extended interval (128-137 ka), SL has been documented at 10-20 m of its highstand position of +3-6 m, from well dated LIG reefs on stable coasts of Western Australia[73,74], Florida[75], the Bahamas[76], Grand Cayman[77], and uplifted Barbados[78,79]. Assuming that the massive *Porites* and tabular acroporid corals in holes 5D and 19A grew in water-depths of 30 m, which is the maximum extent of their modern depth range[19], and a SL that was 10-20 m lower than present at 128 ka, a subsidence rate of 0.5 to 0.6 mm yr^{-1} is required to account for their present elevation at 115-117 m below SL. This rate of subsidence is consistent with the 85 m elevation of the LIG reef-flat platform below the postglacial reef sequence at Maraa site 7, Faaa site 19A, and the Papeete barrier reef. What is clear from this argument, is that resolution of the subsidence rate of Tahiti requires dating of in-situ corals from the uppermost part of the Pleistocene reef-flat platform, as has been standard practice in other areas where postglacial SL has been reconstructed[80].

References for Supplementary Information

56. Blanchon, P. Reef demise and back-stepping during the last interglacial, northeast Yucatan. *Coral Reefs* **29**, 481-498 (2010).
57. Veron, J.E.N. Corals of the world. *Australian Inst. Mar. Sci.*, **vol. 3**, 463 pp (2000).
58. Bromley, R.G., & D'Alessandro, A. Bioerosion in the Pleistocene of southern Italy: ichnogenera Caulostrepsis and Maeandropolydora. *Riv. Ital. Paleont. Stratig.* **89**, 283-309 (1983).
59. Bromley, R.G., & D'Alessandro, A. The ichnogenus Entobia from the Miocene, Pliocene and

Pleistocene of southern Italy. *Riv. Ital. Paleont. Stratig.* **90**, 227-296 (1984).
60. Bromley, R.G., & D'Alessandro, A. Ichnological study of shallow marine endolithic sponges from the Italian coast. *Riv. Ital. Paleont. Stratig.* **95**, 279-314 (1989).
61. Martindale, W. Calcified epibionts as palaeoecological tools: examples from the Recent and Pleistocene reefs of Barbados. *Coral Reefs* **11**, 167-177 (1992)
62. Harwood, G.M. Microscopical Techniques: I. Principles of sedimentary petrography. In: Tucker M.E. (ed) *Techniques in Sedimentology*. Blackwell Scientific Publications, Oxford, pp 108-173 (1988).
63. Blair T.C. & McPherson J.G. Grain-size and textural classification of coarse sedimentary particles. *J. Sediment. Res.* **69**, 6-19 (1999)
64. Hallsworth, C.R. & Knox R.W.O.B. BGS Rock Classification Scheme Vol 3: Classification of sediments and sedimentary rocks. *B.G.S. Res Rep* **RR99-03**, British Geological Survey, Nottingham, UK (1999).
65. Dunham, R.J. Classification of carbonate rocks according to depositional texture. In: Ham, W.E. (Ed.), Classification of carbonate rocks. *Am. Assoc. Pet. Geol. Mem.* **1**, 108-121 (1962).
66. Riding, R. Reef concepts. *Proc. 3rd Intern. Coral Reef Symp., Miami*, pp. 209-213 (1977).
67. Insalaco, E. The descriptive nomenclature and classification of growth fabrics in fossil scleractinian reefs. *Sediment. Geol*. **118**, 159-186 (1988).
68. Riding R. Structure and composition of organic reefs and carbonate mud mounds: concepts and categories. *Earth-Sci. Rev.* **58**, 163-231 (2002).
69. Pandolfi J.M. & Greenstein B.J. Taphonomic alteration of reef corals: effects of reef environment and coral growth form. I. The Great Barrier Reef. *Palaios* **12**, 27-42 (1997).
70. Scholz, D. & Mangini, A. How precise are U-series coral ages? *Geochim. Cosmochim. Acta* **71**, 1935-1948 (2007).
71. Fadil, A., Sichoix, L., Barriot, J.P., Ortega, P. & Willis, P. Evidence for a slow subsidence of the Tahiti Island from GPS, DORIS, and combined satellite altimetry and tide gauge sea level records. *Comptes Rendus Geoscience* **343**, 331-341 (2011).
72. Thomas, A.L. *et al.* Assessing subsidence rates and paleo water-depths for Tahiti reefs using U–Th chronology of altered corals. Mar. Geol. **295-298**, 86-94 (2012).
73. Eisenhauer, A., Zhu, Z.R., Collins, L.B., Wyrwoll, K.H., & Eichstatter, R. The Last Interglacial sea level change: new evidence from the Abrolhos Islands, West Australia. *Geol. Rundsch*. **85**, 606-614 (1996)
74. Stirling, C.H., Esat, T.M., Lambeck, K. & McCulloch, M.T. Timing and duration of the Last Interglacial: evidence for a restricted interval of widespread coral reef growth. *Earth Planet. Sci. Lett.* **160**, 745-762 (1988).
75. Fruijtier, C., Elliott, T. & Schlager, W. Mass-spectrometric 234U- 230Th ages from the Key Largo Formation, Florida Keys, United States: Constraints of diagenetic age disturbance. *Geol. Soc. Am. Bull.* **112**, 267-277 (2000).
76. Chen, J. H., Curran, H. A., White, B. & Wasserburg, G. J. Precise chronology of the last interglacial period: 234U-230Th data from fossil coral reefs in the Bahamas. *Geol. Soc. Am. Bull.* **103**, 82-97 (1991).
77. Vezina, J., Jones, B. & Ford, D. Sea-level highstands over the last 500,000 years; evidence from the Ironshore Formation on Grand Cayman, British West Indies. *J. Sedim. Res.* **69**, 317-327 (1999).
78. Blanchon, P. & Eisenhauer, A. Multi-stage reef development on Barbados during the Last

Interglaciation. *Quat. Sci. Rev.* **20**, 1093-1112 (2001).

79. Gallup, C.D., Cheng, H., Taylor, F.W. & Edwards, R.L. Direct determination of the timing of sea level change during termination II. *Science* **295**, 310-313 (2002).

80. Fairbanks, R.G. A 17,000 year glacio-eustatic sea level record: influence of glacial melting rates on the Younger Dryas event and deep ocean circulation. *Nature* **342**, 637-642 (1989).
